# Pilot test of an accrual Common Metric for the NIH Clinical and Translational Science Awards (CTSA) Consortium: Metric feasibility and data quality

**DOI:** 10.1017/cts.2020.537

**Published:** 2020-09-11

**Authors:** Denise H. Daudelin, Laura E. Peterson, Harry P. Selker

**Affiliations:** 1 Tufts Clinical and Translational Science Institute, Tufts University, Boston, MA, USA; 2 Institute for Clinical Research and Health Policy Studies, Tufts Medical Center, Boston, MA, USA

**Keywords:** Performance improvement, common metrics, translational science, study accrual, CTSA

## Abstract

Failure to accrue participants into clinical trials incurs economic costs, wastes resources, jeopardizes answering research questions meaningfully, and delays translating research discoveries into improved health. This paper reports the results of a pilot test of the Median Accrual Ratio (MAR) metric developed as a part of the Common Metrics Initiative of the NIH’s National Center for Advancing Translational Science (NCATS) Clinical and Translational Science Award (CTSA) Consortium. Using the metric is intended to enhance the ability of the CTSA Consortium and its “hubs” to increase subject accrual into trials within expected timeframes. The pilot test was undertaken at Tufts Clinical and Translational Science Institute (CTSI) with eight CTSA Consortium hubs. We describe the pilot test methods, and results regarding feasibility of collecting metric data and the quality of data that was collected. Participating hubs welcomed the opportunity to assess accrual efforts, but experienced challenges in collecting accrual metric data due to insufficient infrastructure and inconsistent implementation of electronic data systems and lack of uniform data definitions. Also, the metric could not be constructed for all trial designs, particularly those using competitive enrollment strategies. We offer recommendations to address the identified challenges to facilitate progress to broad accrual metric data collection and use.

## Introduction

The accrual of participants is the foundation of clinical studies. Failure to accrue expected or sufficient participants incurs economic costs, wastes resources [1, 2], risks premature closure [3–5], jeopardizes answering the primary research question meaningfully [6,7], and delays the translation of research discoveries into practices that improve health. Moreover, it may result in less research being undertaken as resources are redirected to extending existing trials rather than funding additional studies. Principal investigators (PIs) may be aware of the accrual status of their own trials, but institutions need measures to assess accrual success across their portfolio of trials in order to rationally manage and direct effort and resources where they are most needed [8].

Between January and July 2017, an Accrual Metric Development Team developed the Median Accrual Ratio (MAR) metric and Operational Guideline as a part of the Common Metrics Initiative of the NIH’s National Center for Advancing Translational Science (NCATS) Clinical and Translational Science Award (CTSA) Consortium. The metric development process included an extensive literature review and key informant interviews with stakeholders who had developed similar metrics [9] and/or processes for managing clinical trials accrual data, including the National Cancer Institute (NCI), National Institute of Allergy and Infectious Diseases, and the Rockefeller University Center for Clinical and Translational Science [8]. The Operational Guideline was vetted by a group of CTSA Consortium hub evaluators, PIs, and administrators.

**Fig. 1.**
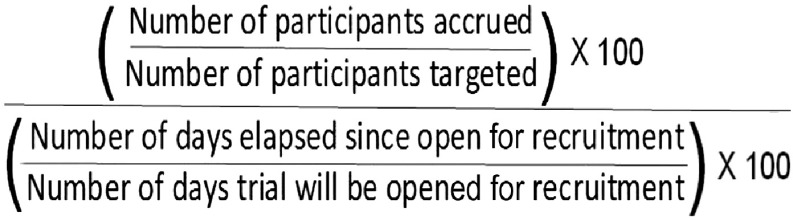
Median Accrual Ratio.

The MAR is designed to be used to enhance CTSA hubs and the CTSA Consortium’s ability to develop performance interventions to increase accrual of participants into trials [10], and is the median across a set of clinical trials of a within-trial ratio (see Fig. 1).

The Common Metrics Implementation Team [11] at Tufts Clinical and Translational Science Institute (CTSI) was asked by NCATS to coordinate a pilot test of the metric to inform future revisions to the metric and its eventual implementation. In this paper, we describe the pilot test methods, and results regarding the feasibility of collecting metric data and the quality of data that could be collected. A companion paper provides results regarding the usefulness of the metric result for conducting strategic management of accrual at CTSA Consortium hubs and nationally [12].

## Methods

### Pilot Test Hub Selection and Characteristics

To identify potential pilot test hubs, Tufts distributed an email solicitation to CTSA hub PIs and staff, asking for volunteers. Among 22 hubs responding, 8 were selected in consultation with NCATS to participate in the 4-month pilot. Selection criteria included expressed interest and staff availability, the institution’s previous experience collecting an accrual metric, and several site characteristics including institution size, use of a fully or partially implemented Clinical Trial Management System (CTMS), and volume and types of clinical trials conducted. Selection was intentionally nonrandom to include a range of site characteristics and capacities for accrual metric data collection and strategic management. After selection, each hub formed a team to participate in the pilot consisting of the PI and other leadership (e.g., an administrator and/or evaluation manager), an evaluator, one or more subject-matter experts on the accrual process (as distinct from an expert on the accrual data or database), a data system expert, and a data analyst. The Tufts Health Sciences IRB determined that the Accrual Metric Pilot Test was no human subjects research.

### Pilot Conduct

Participants received training in the accrual metric Operational Guideline, the Results-Based Accountability strategic management framework used by the Common Metrics Initiative, and the Scorecard software used to document the metric result and a resultant performance improvement plan. In pilot test weeks 3–15, the Implementation Team conducted every other week webinars with each of two groups of hub teams (those from institutions with and without a CTMS). During the alternate weeks, the Implementation Team conducted follow-up calls with individual hubs as needed. Operational Guideline clarifications and responses to hub questions were provided in conjunction with a subgroup of the Accrual Metric Development Team during webinars and through updates to a FAQ document.

### Pilot Test Goal

**Fig. 2.**
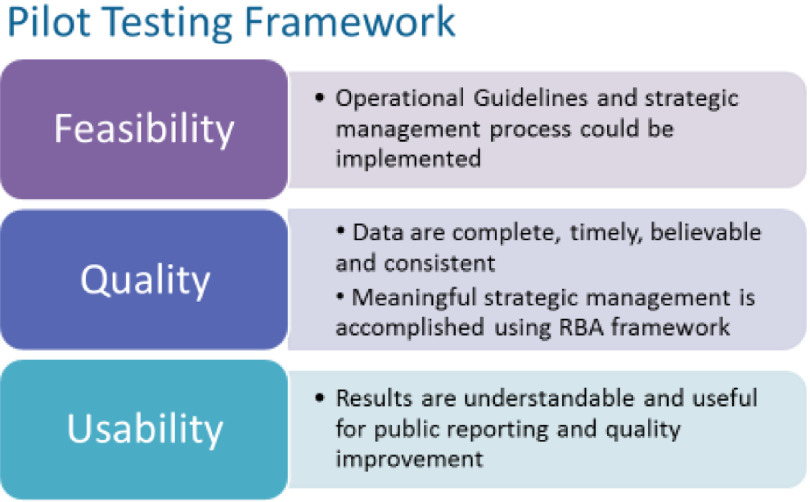
Framework for the Accrual Metric Pilot Test.

The Accrual Metric Pilot Test was designed to assess the feasibility, quality, and usability [13] of the accrual metric for collecting metric data and using it for strategic management (see Framework, Fig. 2). Here, we report results of the assessment of data collection feasibility and quality.

To determine whether data collection was feasible, we assessed hubs’ ability to use existing or obtainable data to construct a metric value following the accrual metric Operational Guideline, and the associated level of effort/burden, considering:

- Whether each pilot hub followed the specification of each section of the Operational Guideline, and any decisions that hubs made due to the underlying structure of their data or other local considerations.
- The extent to which pilot hubs were currently collecting the data elements necessary to create the accrual metric, how readily available the data were, and what manipulation was required in order to match Operational Guideline definitions.
- Relevant hub characteristics, i.e., was data collection more feasible for some groups of hubs than others.
- The nature of any identified barriers, the likelihood of, and timeframe for overcoming them, and any changes to the Operational Guideline that would allow for more immediate or near-term metric collection.

Four components of data quality were considered, including:

- Completeness: Was the metric result reported?
- Timeliness: Was the metric result reported by the time of the pilot test’s completion?
- Believability: Was the metric result within an expected range?
- Consistency: Were data collection procedures and decision rules consistent with the Operational Guideline and across hubs?

### Data Collection

Data were collected from the Tufts Implementation Team notes taken on each of the pilot test webinars, documentation in the Scorecard system, and a post-pilot survey of hub teams. Pilot hubs used the Scorecard system to record their MAR value and describe the sample they used for the metric. One week after the last webinar, a link to a REDCap survey was sent to each hub’s PI and other team members to collect information about the feasibility and quality of data for each hub’s selected sampling frame, the metric inclusion/exclusion criteria, and each of the four variables used to compute the MAR. Survey responses were downloaded from REDCap to Excel for analyses.

## Results

### Pilot Hub Characteristics

Pilot test hub characteristics are shown in Table 1. Pilot hubs with a CTMS varied in the extent of their implementation and types of trials included. Oncology trials were most frequently represented, likely because data were more available due to preexisting NCI reporting requirements.

**Table 1.** Characteristics of hubs (n=8) participating in the Accrual Metric Pilot Test

Characteristics	Number of hubs
CTSA size/funding level	
Small	2
Medium	5
Large	1
Year CTSA first funded	
2006–2007	2
2008–2009	4
2010–2011	2
Approximate annual number of clinical trials at the hub primary institution	
1–100	2
101–250	1
251–500	1
501–1000	3
Greater than 1000	1
CTMS	
None	2
Yes, for some or all clinical trials at the hub primary institution	6
Experience collecting a previous accrual metric	
No	4
Yes, for some clinical trials at the hub primary institution	2
Yes, for all clinical trials at the hub primary institution	2

### Data Collection Feasibility

#### Determining the sampling frame

The MAR metric is intended to apply to all clinical trials at a CTSA hub’s primary institution. However, hubs without a CTMS, or with only a subset of trials using a CTMS, lacked central lists of trials at their institutions to use for their sampling frame. The IRB was a potential source of such a list for some hubs, but IRB data were sometimes difficult to extract from existing electronic or paper systems. Hubs also attempted to build a sampling frame by combining several data sources. Despite these efforts, only one hub (12.5% of the participating sites) was able to identify and collect metric data for all eligible clinical trials at its primary institution. Five (62.5%) of the eight hubs selected nonrandom samples of eligible clinical trials, and the remaining two (25%) conducted random samples.

#### Applying inclusion/exclusion criteria

*Ability to determine inclusions/exclusions*: The Operational Guideline specified inclusion/exclusion criteria for trials included in calculating the MAR. Although most hubs had limited their sampling frame, they were still not able to determine inclusion/exclusion criteria for all of the trials in the sample (Table 2).

**Table 2.** Ability to determine whether trials met inclusion/exclusion criteria from the data source(s) used for the sample (n = 8)

Inclusion/exclusion criteria	Able to determine inclusion/exclusion criteria for *all* of the trials in the sample	Able to determine inclusion/exclusion criteria for *some* of the trials in the sample	Not able to determine inclusion/exclusion criteria
Trials met the NIH clinical trial definition	5 (62.5%)	1 (12.5%)	2 (25%)
Trials required informed consent	6 (75%)	1 (12.5%)	1 (12.5%)
Trials had less than 10 targeted participants	5 (62.5%)	3 (37.5%)	0 (0%)
Dose escalation to toxicity trials could be identified	3 (37.5%)	2 (25%)	3 (37.5%)

*Trials with fewer than 10 targeted participants*: For purposes of the pilot test, trials with less than 10 targeted participants were excluded to avoid potentially skewing metric values by including very small trials with very low expected accrual. Among the seven hubs that could estimate the effect of this exclusion on their sample of eligible trials, an average of 38.7% of clinical trials (median 32%; range 9%–74%) were excluded because they had fewer than 10 targeted participants or the number of targeted participants were unknown.

*Additional trial exclusion*: After the pilot test began, hubs were instructed to exclude dose-escalation trials due to concern that the trial design precluded accurate estimation of the number of planned targeted participants. Almost two-thirds of pilot hubs (5/8; 63%) reported that data were not available to evaluate this criterion for some or all of the trials in their sample.

#### Data sources for key metric variables

Despite significantly limiting the number of trials in their sampling frames, only three of the five hubs with a full or partial CTMS were able to create the metric using only the CTMS (Table 3). Five hubs required multiple sources (range 3–6) to collect data for the four key metric variables for the trials in their sample. Hubs who could not obtain all metric data from one system used additional systems or collected new primary data. Once data were collected, multiple datasets needed to be assessed, merged, and cleaned.

**Table 3.** Data sources used to collect data elements in the hub sample

Hub	CTMS	Non-CTMS electronic system (e.g., eIRB)	IRB progress reports	Study Protocol	REDCap Survey	Email or phone call	Other	Total data sources (N)
A	X							1
B	X							1
C	X	X	X	X	X	X		6
D	X							1
E	X						X*	3
F		X	X	X	X			4
G		X	X	X	X	X		5
H			X		X		X	3

* Hub used two different other data sources.

#### Barriers to collecting key metric variables

Hubs reported data collection barriers for all four variables, even when using multiple data sources (Table 4). The reported barriers varied considerably by hub, data source(s) used, and metric variable.

**Table 4.** Number and percentage of hubs reporting barriers to data collection for four key metric variables

MAR variable	Reported barriers to collecting	
	Yes (N, %)	No (N, %)
Number of participants accrued	3 (37.5%)	5 (62.5%)
Number of participants targeted	4 (50%)	4 (50%)
Date open for recruitment	4 (50%)	4 (50%)
Number of days planned to be opened for recruitment	7 (87.5%)	1 (12.5%)

*Number of participants accrued*: Hubs with a CTMS varied in their ability to determine the number of participants accrued for a particular timeframe. Influencing factors included the extent to which trials at the primary institution used the CTMS, and the system’s reporting capabilities. One hub’s CTMS lacked a ready way to automatically generate a report of a number accrued as of some specific date in the past rather than the accrual to date. Hubs without a CTMS attempted to determine this value via a survey or electronic data system maintained for other purposes. Although instructions were included at the four hubs that used a survey, trial investigators and/or their staff were not always clear about which participants they should report.

*Number of participants targeted*: At half of the pilot hubs, this variable was a barrier for some or all clinical trials in the sampling frame. In several instances, it was not a field in the hub’s CTMS, or was present but specified differently than in the Operational Guideline. Hubs without a CTMS typically used an eIRB database or a survey to collect the number of participants targeted.

*Number of days elapsed since open to recruitment*: Calculating this variable requires the date that the trial opened to recruitment. For half of the pilot hubs, determining the date a trial opened was a barrier for some or all clinical trials in their sample. Availability in the CTMS varied. Non-CTMS hubs relied on survey data for this variable.

*Number of days trial will be open to recruitment*: This was the most problematic of the variables. Seven out of eight pilot hubs reported barriers to collecting it for some or all trials in their sample. For hubs with a CTMS, the variable was not present or not specified as per the Operational Guideline. The planned recruitment duration was reportedly often unknown by the study team, or considered, at best, a guess. For hubs without a CTMS, the number of days planned to be open to recruitment was not available in preexisting databases.

#### Survey considerations

Four of the eight (50%) pilot hubs conducted a REDCap survey to collect and/or verify at least one data element or inclusion/exclusion criterion. Logistic challenges to fielding the surveys included not knowing who should receive the request to complete the survey, and devising instructions to ensure that responses were consistent with Operational Guideline definitions. Surveys remained open for a limited period of time as considerable time had been expended planning and fielding the surveys. It is possible that response rates could exceed the 39%–60% achieved by the pilot hubs if surveys are open longer.

#### Facilitators of data collection

Among sites without a fully implemented CTMS, the most commonly identified potential facilitator for data collection was institution-wide adoption of a single CTMS. Mandating or promoting accrual data entry into this CTMS was perceived to be a key factor. Respondents also stressed the importance of obtaining buy-in from various stakeholders and forming collaborative relationships with them. Stakeholders played important roles during the pilot such as by providing access to existing data sources and helping with the collection of new data. For sites that collected data on cancer trials, the NCI mandate to collect accrual data meant there was already an electronic data collection infrastructure in place as well as routine data entry by study teams. Among the six hubs reporting use of a CTMS for some or all trials at their primary institution (Table 1), four indicated that cancer-related trials utilized the CTMS (although not all of these hubs necessarily had NCI designation). Other practices at cancer centers that respondents found helpful were routine data cleaning and having a committee to vet a study team’s initial recruitment target.

#### Number of trials in the MAR

After applying sampling frames and inclusion/exclusion criteria, and removing trials with missing or incomplete data (including survey nonresponse), the mean number of clinical trials included in the MAR across pilot hubs was 76.1 (median = 57.5; range 6–212). In 3 of the 8 hubs (37.5%), the MAR was calculated based on fewer than 20 eligible clinical trials.

### Data Quality

All pilot hubs encountered data quality challenges when collecting accrual data for the pilot, including missing or incomplete data, inconsistencies or conflicting data, and potential survey bias. Data conflicts often arose when variables were obtained across multiple data sources that utilized inconsistent definitions for the “same” field (e.g., yielding different values for the number of participants accrued across multiple electronic databases). Preexisting definitions in hub data sources often did not match the definitions provided in the accrual metric Operational Guideline.

Hubs that used a CTMS for the pilot faced many of the same data challenges as did hubs that did not have a CTMS, as their CTMS data entry requirements were not designed to align with the Operational Guideline. Additional challenges for CTMS hubs included unstructured data that needed to be converted to discrete data, inconsistencies in the use of definitions by different study teams, and inconsistencies in values reported by different members from the same study team. When CTMS or other existing data systems were not already collecting required data, sites used proxy variables (e.g., projected length of study vs. projected days of recruitment), removed trials from the sample, or collected new primary data (e.g., PI survey). Using a proxy variable compromised the accuracy of the metric result, and removing trials from the sample undermined the representativeness of the metric result.

Even when present, hubs found certain values not believable, particularly for the number of days planned to be open to recruitment and the number of targeted participants. For example, multiple hubs expressed considerable skepticism about the accuracy of data from IRB sources on the number of participants targeted. There was a sense that PIs overestimate in order to avoid the need for future IRB amendments to increase their sample sizes. Hubs also reported a lack of confidence as to whether PIs and study teams are reliably able to determine whether a study is a clinical trial. One hub compiled a list of studies that seemed to meet the definition of a clinical trial based on IRB data; however, when surveyed, many study teams reported that their study was not a clinical trial. Additional survey-specific data quality issues included wrong email addresses and investigators. In instances where only the IRB number or protocol number was available, some study staff or investigators were not able to identify a trial without its title.

### Feasibility of Expanding the Sample

Most hubs reported that their pilot data collection approach would not be sustainable due to the great level of effort required, the low confidence in the data collected, and the diversion of effort from other important CTSA areas and strategic management for accrual. Many respondents worried that expanding data collection to all eligible trials would be a “big mandate” that may not be feasible for at least 2–3 years, and some believed it would require a long, complex planning process.

Five of the six hubs without broad CTMS implementation reported advocating for or planning to implement an institution-wide CTMS, and expected increased feasibility of expanding their sample to additional clinical trials in the future.

## Discussion

Accrual metrics offer the opportunity for investigators, research organizations, and research networks such as the CTSA Consortium to measure and improve the performance of clinical trials. We conducted a pilot test with eight CTSA Consortium hubs to determine the feasibility of collecting accrual metric data and the quality of data that could be collected. All hubs completed the pilot including selecting a sampling frame, attempting to apply the inclusion and exclusion criteria, identifying data sources, and collecting metric variables. This pilot test revealed challenges in collecting the accrual metric due to insufficient infrastructure including electronic data systems, inconsistent implementation of those systems when they were available, and lack of uniform or harmonized data definitions. Further, although intended to apply broadly across clinical trials, the metric could not be constructed for all trial designs, particularly some that use competitive enrollment strategies. Therefore, we offer several recommendations to address the identified challenges.

**Allow for an infrastructure-building period prior to mandated accrual metric data collection:** The organizational infrastructure needed for broad implementation of the accrual metric includes data collection and reporting systems deployed across all clinical trials, using uniform data definitions, and emphasizing data quality. Existing data sources do not currently align with the inclusion/exclusion criteria and variable definitions in the accrual metric Operational Guideline, or lack key variables entirely. To implement ongoing data collection, hubs will need to devise and/or revise data sources and systems and data collection procedures, institute data cleaning processes, and train personnel. Some hubs may benefit from leveraging their existing cancer center resources and stakeholders to build the needed infrastructure.

**Provide hubs with best or promising practices and strategies for implementing a CTMS to produce metrics**: Implementing a CTMS was believed to improve the feasibility of collecting the accrual metric, although not all hubs with a CTMS were able to produce the metric data. Since many CTSA Consortium hubs are planning new CTMS implementations, or modifications to existing systems, the timing is auspicious to incorporate guidance on metric data requirements into system builds and user training.

**Provide a template of tested survey questions and survey considerations**: Developing survey questions, definitions and procedures was time-consuming for hubs without a CTMS who collected data using surveys.

**Address clinical trial designs in which key MAR variables are not known**: Certain clinical trial designs, including dose-escalation trials, competitive enrollment strategies, and trials with planned small accrual targets, have potential implications for availability of metric data, and whether inclusion of such trials may bias or skew metric results. In this pilot test, most hubs were unable to operationalize excluding dose-escalation trials without eliminating all Phase I trials. Further, the rationale that accrual is “different” for subjects in these types of trials is uncertain. Therefore, we recommend that dose-escalation trials not be excluded from the accrual metric. Sponsors and coordinating centers of some multisite trials using a competitive enrollment design do not provide the targeted number of participants and/or the planned recruitment period to an individual site. Even when these metric variables are known initially, they may change over time if the overall study accrual target is reached and the recruitment period is cut short. We recommend that analysis of a larger data set of trials be evaluated to assess the potential effects on the MAR of excluding trials with competitive enrollment designs. Finally, excluding trials with low accrual targets removes many otherwise potentially eligible clinical trials from the sampling frame, particularly at small primary institutions with smaller pools of clinical trial participants from which to draw, or those institutions with large numbers of multisite trials for which they are recruiting a subset of participants. We, therefore, recommend that the exclusion criterion for trials with less than 10 targeted participants be reevaluated.

### Outstanding Issues

In addition to these recommendations, areas remain for which solutions are needed. Although we recommend an infrastructure-building period prior to requiring metric data collection, clear best practices for such a system that can systematically and consistently address data quality do not yet exist. Therefore, building an effective infrastructure will take time and resources and will itself require an iterative improvement process. Any effective infrastructure must ensure data can be collected without requiring excessive resources that preclude using the data for improvement. There is also inherent tension in the attempt to align accrual metric Operational Guideline data definitions with other CTMS operational needs, as these systems are implemented to support metric reporting efforts beyond the Common Metrics, and functions besides metric reporting. Despite the challenges, there are important opportunities for learning. As organizations revise their existing data systems and implement new processes, they are likely to uncover valuable insights that can and should be shared systematically across the CTSA Consortium.

## Conclusion

In this pilot test of the Common Metric MAR, all participating CTSA Consortium hubs found the process of examining the feasibility and quality of their existing accrual data helpful. However, significant challenges remain in the feasible collection of high-quality data with which to assess the current accrual ratio for clinical trials at the hub and CTSA Consortium levels.

## References

[1] Stensland KD , et al. Adult cancer clinical trials that fail to complete: an epidemic? Journal of the National Cancer Institute 2014; 106(9): dju229. 10.1093/jnci/dju229 25190726

[2] Rimel BJ. Clinical trial accrual: obstacles and opportunities. Frontiers in Oncology 2016; 6: 103. 27200292 10.3389/fonc.2016.00103 PMC4843106

[3] Schroen AT , et al. Preliminary evaluation of factors associated with premature trial closure and feasibility of accrual benchmarks in phase III oncology trials. Clinical Trials 2010; 7(4): 312–321. 20595245 10.1177/1740774510374973 PMC3977321

[4] Baldi I , Lanera C , Berchialla P , Gregori D. Early termination of cardiovascular trials as a consequence of poor accrual: analysis of ClinicalTrials.gov 2006–2015. BMJ Open 2017; 7(6): e013482. 10.1136/bmjopen-2016-013482 PMC5577897 28619765

[5] Bernardez-Pereira S , et al. Prevalence, characteristics, and predictors of early termination of cardiovascular clinical trials due to low recruitment: insights from the ClinicalTrials.gov registry. American Heart Journal 2014; 168(2): 213–219.e1. 25066561 10.1016/j.ahj.2014.04.013

[6] Carlisle B , Kimmelman J , Ramsay T , MacKinnon N. Unsuccessful trial accrual and human subjects protections: an empirical analysis of recently closed trials. Clinical Trials 2015; 12(1): 77–83. 25475878 10.1177/1740774514558307 PMC4516407

[7] Schroen AT , et al. Achieving sufficient accrual to address the primary endpoint in phase III clinical trials from U.S. Cooperative Oncology Groups. Clinical Cancer Research 2012; 18(1): 256–262. 21976533 10.1158/1078-0432.CCR-11-1633 PMC3977198

[8] Corregano L , Bastert K , Correa da Rosa J , Kost RG. Accrual Index: a real-time measure of the timeliness of clinical study enrollment. Clinical Translational Science 2015; 8(6): 655–661. 26573223 10.1111/cts.12352 PMC4703441

[9] Rubio DM , et al. Developing common metrics for the Clinical and Translational Science Awards (CTSAs): lessons learned. Clinical Translational Science 2015; 8(5): 451–459. 26073891 10.1111/cts.12296 PMC4626292

[10] CTSA Program Common Metrics Operational Guideline: Median Accrual Ratio [Internet]. National Center for Advancing Translational Sciences. 2019 [cited 2020 Apr 15]. Available from: (https://clic-ctsa.org/sites/default/files/2019-09/Median%20Accrual%20Ratio%20Metric%20Operational%20Guidelines_6.pdf)

[11] Daudelin DH , et al. Implementing Common Metrics across the NIH Clinical and Translational Science Awards (CTSA) consortium. Journal of Clinical and Translational Science 2020; 4(1): 16–21. 32257406 10.1017/cts.2019.425 PMC7103469

[12] Peterson LE , Daudelin DH , Selker HP. Pilot test of an accrual Common Metric for the NIH Clinical and Translational Science Awards (CTSA) Consortium: Metric usefulness. Journal of Clinical and Translational Science. doi: 10.1017/cts.2020.544. PMC8057423 33948271

[13] Adapted from: Measure Evaluation Criteria and Guidance for Evaluating Measures for Endorsement. National Quality Forum; 2016 Aug.

